# Efficacy and safety of an implantable tibial neuromodulation system for overactive bladder with urgency urinary incontinence: an open-label, single arm trial

**DOI:** 10.3389/fruro.2026.1865760

**Published:** 2026-07-15

**Authors:** Fuyuan Zheng, Yongjun Guan, Weilin Fang, Tingting Lv, Jin Huang, Xin Song, Jianwei Lv

**Affiliations:** 1 School of Gongli Hospital Medical Technology, University of Shanghai for Science and Technology, Shanghai, China; 2 Pudong Gongli Hospital, Shanghai University of Medicine & Health Sciences, Shanghai, China

**Keywords:** efficacy, overactive bladder, safety, tibial nerve stimulation, urgency urinary incontinence

## Abstract

**Purpose:**

This study aimed to evaluate the safety and efficacy of a domestically developed implantable tibial neuromodulation system (iTNS) in the treatment of overactive bladder (OAB) in patients with urgency urinary incontinence (UUI).

**Methods:**

A prospective single-center open-label trial was conducted. A total of 10 participants underwent iTNS device implantation, followed by device activation at 2–4 weeks post-implantation. Follow-up evaluations were performed at 1, 3, and 6 months post-implantation, including 3-day bladder diaries, validated questionnaires, and symptom satisfaction surveys. The primary endpoint was a ≥50% reduction in UUI episodes per day at 6 months.

**Results:**

At 6 months, the iTNS system achieved a 90% response rate (9/10 patients), among whom 40% (4/10) attained ≥3 consecutive dry days. A delayed therapeutic onset was observed: a significant reduction in UUI episodes emerged at 3 months (from 3.235 to 0.766 episodes per day) and 6 months (from 3.235 to 0.632 episodes per day). Quality of life was significantly improved at 6 months, as indicated by the changes in validated questionnaires (OAB-Q: 69.5 ± 11.76 to 44.4 ± 12.06; OABSS: 9 ± 1.155 to 6.2 ± 2.394). Additionally, 80% of patients reported satisfaction with their symptoms at the 6-month endpoint.

**Conclusions:**

The domestically developed iTNS system is safe and effective for the treatment of UUI in patients with OAB. It exhibits a strong ability to significantly reduce or completely resolve UUI symptoms, with a favorable safety profile.

## 1 Introduction

Overactive bladder (OAB) is a clinical syndrome characterized by urinary urgency, with or without urgency urinary incontinence (UUI), often accompanied by increased urinary frequency and nocturia. It is a highly prevalent lower urinary tract dysfunction with a reported prevalence ranging from 1.5% to 36.4% globally (1). OAB can be diagnosed in the absence of urinary tract infection or other identifiable pathologies. Approximately one-third of patients with OAB also present with UUI, which significantly impairs quality of life, leading to social withdrawal, psychological distress, and reduced daily activity levels (2).

OAB treatment follows a step-wise approach. First-line behavioral approaches, including bladder training, fluid management and pelvic floor exercises, have limited long-term efficacy and poor patient adherence due to the need for sustained self-discipline. Second-line pharmacotherapy, consisting of anticholinergic agents and β3-agonists, is commonly associated with systemic side-effects such as dry mouth, constipation, and cognitive dysfunction, which further reduce treatment compliance, especially in elderly patients. For patients who continue to suffer from intractable symptoms despite conventional first-line and second-line treatments, third-line treatment options include intravesical botulinum toxin injection, sacral neuromodulation (SNM), or tibial nerve stimulation (TNS).

However, these third-line treatments have their own limitations. Although SNM can be effective for the most symptomatic patients, it is costly and involves invasive procedural steps such as sacral lead placement. Additionally, SNM is associated with device-related adverse events like pain, infection, and lead migration, as well as requiring regular long-term follow-up and programming adjustments (2). Intravesical botulinum toxin injection, while effective in reducing symptoms, requires repeated injections every 3–6 months and carries the risk of urinary retention, which can be particularly problematic in patients with impaired bladder emptying function.

Tibial neuromodulation (TNS) is a minimally invasive alternative for managing OAB, with multiple randomized controlled trials (RCTs) demonstrating its efficacy. In the landmark SUmiT trial (N = 220), 54.5% of patients receiving percutaneous tibial nerve stimulation (PTNS) demonstrated moderate to significant symptom improvement, compared to 20.9% in the sham group (3). The OrBIT trial showed comparable efficacy between PTNS and extended-release tolterodine, while PTNS demonstrated a more favorable safety profile (4). Meta-analyses further confirm that TNS significantly reduces incontinence episodes and improves quality of life (5), making it a promising third-line option for refractory OAB.

Traditional TNS modalities, including percutaneous (PTNS) and transcutaneous (TTNS) stimulation, necessitate repeated clinic visits, which can be burdensome for patients and may reduce long-term adherence. Implantable TNS (iTNS) systems address these limitations by offering continuous, patient-controlled neuromodulation, eliminating the need for frequent clinic attendance. Currently, two implantable TNS devices have received FDA approval: the eCoin™ (Valencia Technologies) (6) and the Revi™ system (previously RENOVA iStim, BlueWind Medical) (7). However, there is a lack of clinical data on domestically developed iTNS systems in Chinese patient populations, which may have distinct anatomical and clinical characteristics compared to Western cohorts.

This study investigates the safety and efficacy of a domestically developed implantable tibial nerve stimulator (Microport) in Chinese patients with refractory OAB. The system adopted in this research has several innovations: (1) a two-way visual programmer presenting real-time parameters and battery status, in contrast to the one-way programmer of the eCoinTM; (2) a lead-less ultra-compact system with lower volume and board layout occupancy; (3) high-efficiency power circuitry to achieve a longer battery lifespan; and (4) automated operation that delivers therapy according to pre-set parameters after initial activation, eliminating the need for frequent hospital visits for reprogramming sessions. Herein, we report the first clinical evaluation of this domestically developed iTNS system in a Chinese patient group.

## 2 Patients and methods

### 2.1 This was a prospective, exploratory, first-in-human pilot study

This prospective, single-arm, open-label study was conducted in accordance with China NMPA regulations and Good Clinical Practice guidelines. The study protocol was approved by the Institutional Review Board (IEC-C-008-A007-V1.0) and registered at ChiCTR2300078571, and all methods were performed in accordance with the relevant guidelines and regulations. Written informed consent was obtained from all patients prior to enrollment.

### 2.2 Device description and implantation procedure

The implantable tibial nerve stimulator features an integrated cylindrical generator (diameter 20.2 mm, thickness 4.8 mm) containing both the neurostimulation circuitry and battery within a biocompatible titanium shell. Therapy activation and parameter adjustment are performed wirelessly via an external programmer. All implantation procedures were completed under local subcutaneous anesthesia.

A 1.5–2 cm longitudinal skin incision was made approximately 3 cm cephalad to the medial malleolus of the ankle. Blunt dissection through subcutaneous fat was performed to reach the deep fascia overlaying the posterior tibial neurovascular bundle. The tibial nerve trunk was not fully dissected and anatomically exposed; intraoperative wireless electrical stimulation delivered by the prototype IPG was applied to identify the target nerve. Positive localization was confirmed by two consistent responses: plantar cutaneous paresthesia (sensory response) and involuntary toe flexion (motor response). Stimulation intensity was titrated intraoperatively to avoid excessive painful paresthesia.

After successful nerve localization, a suitable subcutaneous pocket was bluntly dissected 0.5–1 cm adjacent to the tibial nerve trunk. The cylindrical IPG was fully embedded into the pocket, and two absorbable polyglactin sutures were used to fix the titanium device capsule onto deep fascia to prevent postoperative device migration. Real-time stimulation testing was repeated after fixation to verify stable sensory-motor responses before layered closure of subcutaneous tissue and skin.

**Figure 1:**
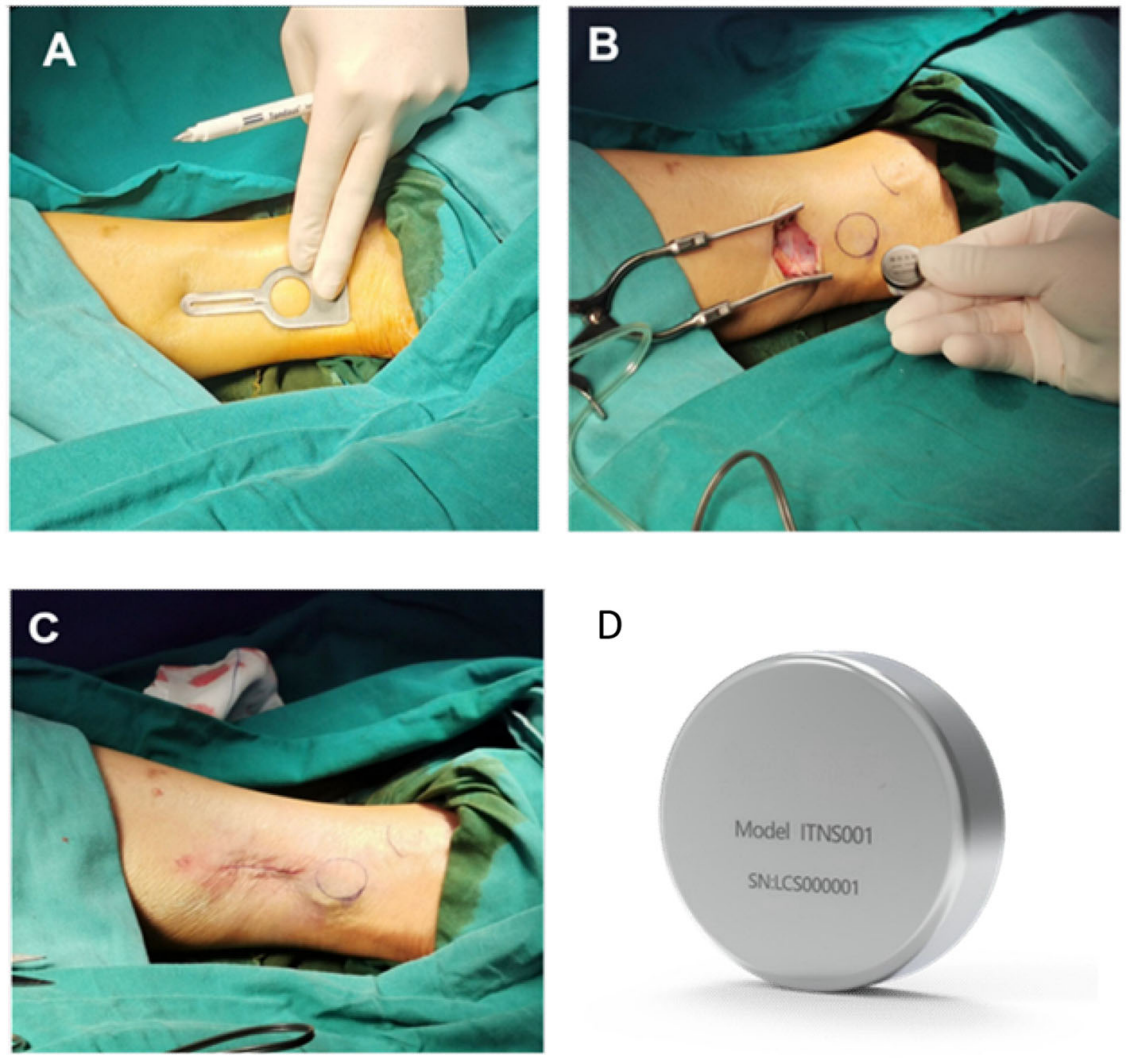
Domestic implantable tibial neuromodulation system and intraoperative implantation procedures. **(A)** Intraoperative anatomical localization of posterior tibial nerve; **(B)** Implantation and fixation of the pulse generator; **(C)** Suture closure of surgical incision; **(D)** Physical diagram of the domestically developed lead-less iTNS device.

For obese patients with thick subcutaneous adipose tissue, surgical protocols were adjusted correspondingly: incision length was extended to 2.0–2.5 cm, the subcutaneous pocket was dissected 1–1.5 cm deeper to maintain sufficient proximity between the IPG and tibial nerve, and extra fascia sutures were applied for reinforced fixation to counteract soft tissue mobility-induced device displacement (**Figure 1**).

### 2.3 Postoperative management and follow-up

Device activation occurred 2–4 weeks post-implantation. During this session, stimulation parameters were individually titrated via the external programmer to achieve optimal symptom relief without discomfort. Following activation, the device operates automatically according to pre-programmed settings. Follow-up visits were scheduled at 1-, 3-, and 6-months post-implantation. At each visit, patients completed a 3-day bladder diary and the Overactive Bladder Questionnaire (OAB-Q) and Overactive Bladder Symptom Score (OABSS). Adverse events, stimulation efficacy, and device performance were documented throughout the study.

### 2.4 Stimulation parameters

Stimulation amplitude ranges from 0.5 to 15 mA, stimulation frequency ranges from 5 to 40 Hz, and stimulation pulse width ranges from 100 to 1, 000 μs. The default stimulation parameters upon startup are set to 6 mA, 20 Hz, and 200 μs. After startup, the stimulation amplitude is typically adjusted to the minimum threshold at which the patient can perceive the stimulation, while the frequency is commonly set to 12.5, 15, or 20 Hz, and the pulse width is usually set to 200 or 300 μs.

### 2.5 Primary efficacy endpoints

The primary efficacy endpoints were evaluated at 1- and 3-months post-activation from baseline as follows: number of urge urinary incontinence episodes per 24 hours, number of voiding episodes per 24 hours, and number of urgency episodes per 24 hours based on 3-day bladder diaries. A responder was defined as a patient with a ≥50% reduction in UUI episodes or urgency episodes from baseline.

### 2.6 Quality of life outcomes

The patients were assessed with the validated Overactive Bladder Questionnaire (OAB-Q) and Overactive Bladder Symptom Score (OABSS) instruments, which offered an assessment of both the severity of symptoms as well as the impact on patient well-being. Lower OAB-Q and OABSS scores indicate better quality of life and fewer symptoms, respectively.

### 2.7 Patient satisfaction

Post-implantation patient satisfaction surveys were performed to assess treatment acceptability as well as evaluate patient-reported outcomes beyond the standard clinical endpoints. Patients were asked, “Compared to before implantation, how would you rate the comfort of wearing the device?” and “Compared to before implantation, how would you rate the improvement in your OAB symptoms?” For both questions, responses were recorded on a 6-point ordinal scale: ‘Extremely good/Very good/Somewhat good/About the same as before/Much worse than before/Extremely poor now’. For analysis, satisfaction for each domain was defined as a positive response. Specifically, patients selecting “Extremely good, “ “Very good, “ or “Somewhat good” were classified as “Satisfied.” This method provides a patient-centric, global assessment of both therapeutic outcome and device tolerability.

### 2.8 Safety endpoints

Safety was evaluated by monitoring device-related and procedure-related adverse events systematically throughout the 6-month follow-up period, assessing both immediate post-operative adverse events as well as device-associated adverse events arising in the long-term. Adverse events were classified according to severity and relationship to the device or procedure.

### 2.9 Statistical analysis

All statistical analyses were performed using GraphPad Prism version 9.0. Continuous variables (voiding diary parameters and questionnaire scores) were expressed as mean ± standard deviation (SD). Baseline versus follow-up variables were compared using one-way ANOVA with repeated measures. P<0.05 was considered statistically significant. Missing data were dealt with using the last observation carried forward (LOCF) method.

## 3 Results

### 3.1 Patient demographics and baseline characteristics

Baseline demographic, OAB disease duration, previous treatment history, comorbidities and lifestyle data of all 10 female participants are summarized in **Table 1**. The standardized inclusion and exclusion screening criteria for this trial are listed in **Table 2**. A cohort of ten female patients (mean age 53.7 ± 12.74 years; range 26-75) with refractory OAB were successfully implanted and activated with the tibial neuromodulation system. One patient dropped out of follow-up at the 6-month timepoint, and the missing value was replaced with the last observation carried forward (LOCF). Comprehensive baseline assessments and a 90% retention rate among 9 completers (n=9/10) indicated high protocol compliance. Baseline characteristics, including UUI episodes, urgency episodes, voiding frequency, and questionnaire scores, were consistent with those of typical refractory OAB patients, confirming the representativeness of the study cohort.

**Table 1:** Baseline demographic of the study cohort.

Characteristic	Value
Age (years)	53.7 ± 12.74 (range 26–75)
Gender, n (%)	Female, 10 (100%)
Duration of OAB, n (%)	<1 year: 2 (20%) 1–2 years: 7 (70%) 2–5 years: 1 (10%) >5 years: 0 (0%)
Previous conservative therapies, n (%)	Bladder training: 7 (70%) Oral anticholinergic/β3 agonist medication: 10 (100%) Intravesical botulinum toxin injection: 0 (0%) Percutaneous tibial nerve stimulation (PTNS): 0 (0%)
Comorbidities, n (%)	Hypertension: 5 (50%) Type 2 diabetes mellitus: 2 (20%) Hyperlipidemia: current 2 (20%), cured 1 (10%) Coronary heart disease: 1 (10%)
Lifestyle history, n (%)	Smoking: never smoked 8 (80%), former smoker 2 (20%) Alcohol intake: never drinker 8 (80%), current drinker 1 (10%), former drinker 1 (10%)

**Table 2:** Patients selection criteria.

Category	Criteria
Inclusion criteria	1. Female patients aged 18–80 years 2. Idiopathic OAB symptoms (urgency, frequency, UUI) lasting ≥6 months 3. ≥8 voids per 24 h confirmed by 3-day bladder diary 4. Persistent symptoms after single-line anticholinergic or β3 agonist treatment
Exclusion criteria	1. Neurogenic bladder, bladder outlet obstruction, urologic malignancy 2. History of urinary retention or ≥3 recurrent UTIs per year 3. Pregnant or planned pregnancy within 12 months 4. Contraindications to local anesthesia or implanted electrical devices

### 3.2 Primary efficacy outcomes

**Figure 2:**
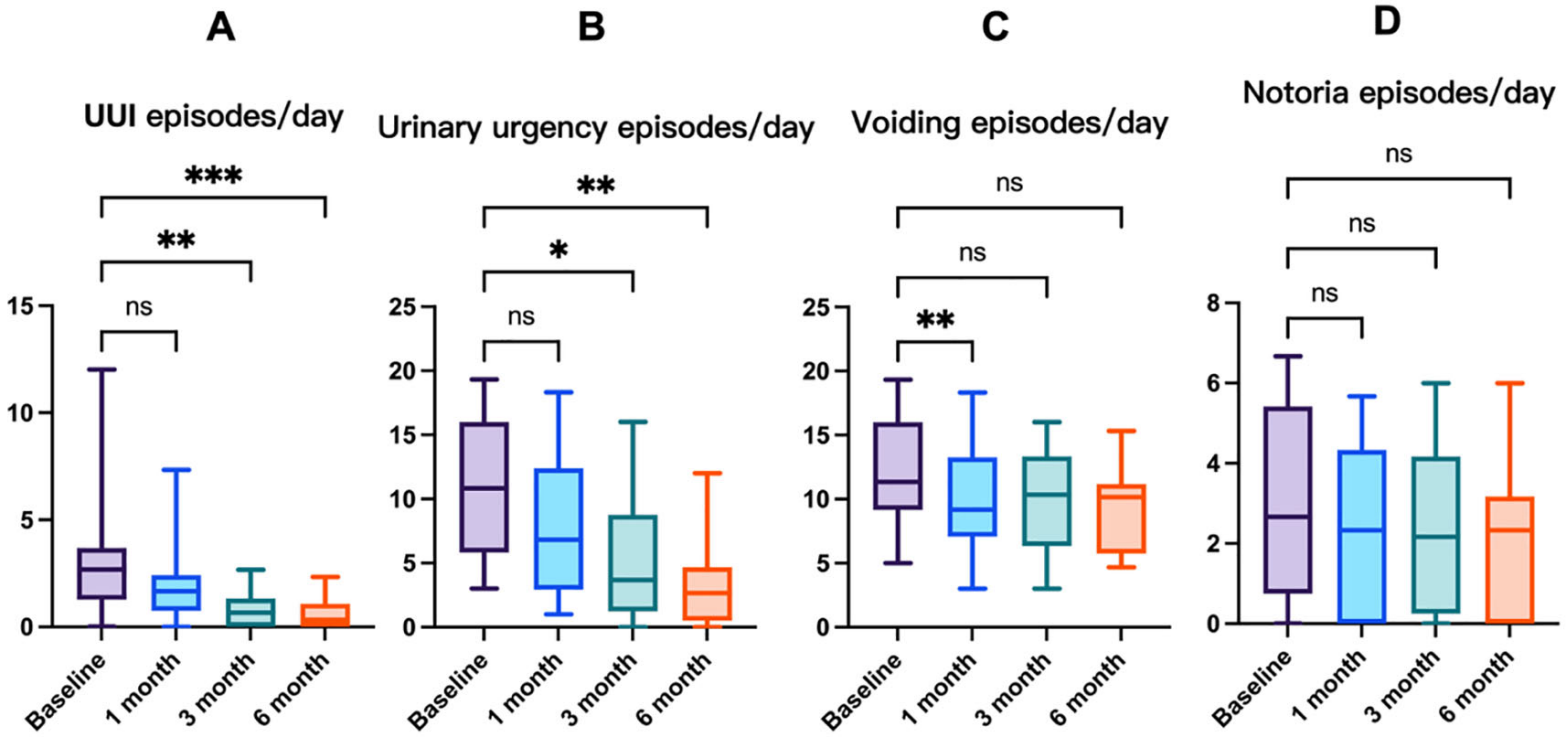
Voiding diary parameters: **(A)** Daily urinary urgency incontinence (UUI) episodes significantly decreased at both 3 and 6 months after activation; **(B)** daily urgency episodessignificantly decreased at both 3 and 6 months after activation; **(C)** voiding frequency significantly decreased at 1 month; **(D)** no significant changes were noted in the number of nocturia episodes at any timepoint.

**Figure 3:**
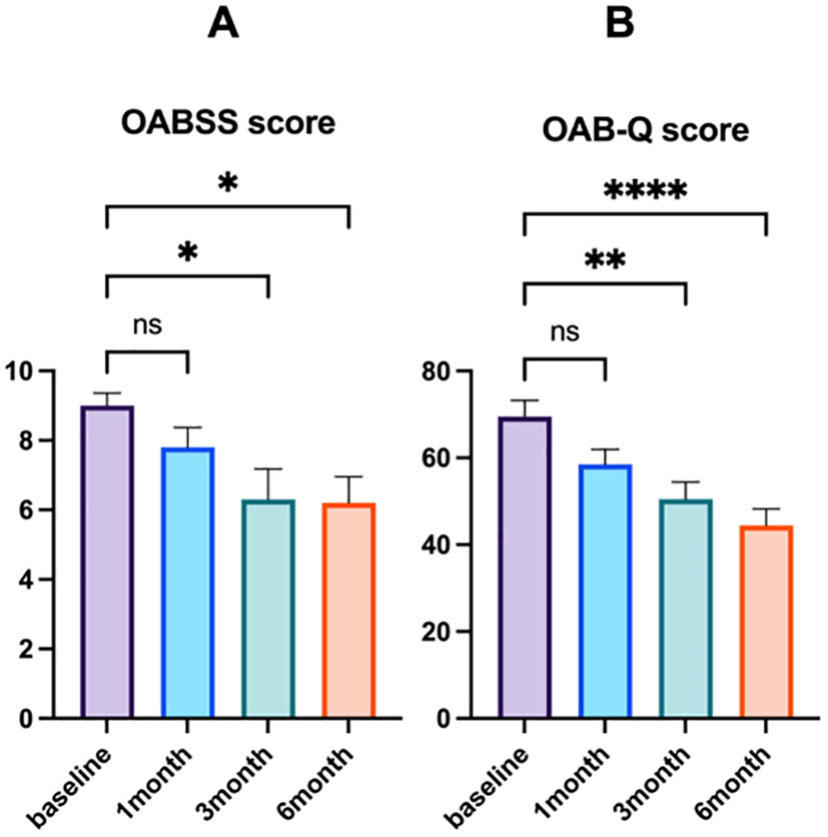
OAB-Q and OABSS questionnaires were significantly improved at 3 and 6 months. Bar graph with two panels labeled **(A, B)** compares OABSS and OAB-Q scores at baseline, 1 month, 3 months, and 6 months. Both scores decrease over time. Statistical significance is indicated by asterisks, with higher significance observed in panel **(B)**.

Clinically, the responder rate (≥50% reduction in UUI episodes) reached 50% at 1 month, 70% at 3 months, and 90% at the 6-month endpoint. For daily urgency episodes, the responder rate (≥50% reduction from baseline) was 40% at 1 month, 70% at 3 months, and 80% at the 6-month endpoint. Analysis of voiding diary parameters indicated different patterns of therapeutic response over time. Daily urinary urgency incontinence (UUI) episodes, which were not normally distributed, significantly decreased at both 3 (p=0.0097) and 6 months (p=0.0008) after activation (Kruskal-Wallis p< 0.05; **Figure 2**). At 1 month, there was no significant decrease in daily UUI (Kruskal-Wallis p> 0.05). Similarly, daily urgency episodes (normally distributed) did not significantly decrease at 1 month (ANOVA p> 0.05; **Figure 3**). Significant decreases were noted at all other timepoints (ANOVA p< 0.05; baseline vs 3 months: p=0.0418, 95% CI [0.173, 10.96]; baseline vs 6 months: p=0.004, 95% CI [2.206, 12.99]). Voiding frequency significantly decreased at 1 month (ANOVA p< 0.05, p=0.0087, 95% CI [0.5174, 3.083]), but this significance did not persist at other timepoints. No significant changes were noted in the number of nocturia episodes at any timepoint (ANOVA p> 0.05; **Figure 2**).

### 3.3 Patient-reported outcomes

Patient-reported outcomes showed a similar delayed onset of therapeutic effect as voiding parameters. For both OAB-Q and OABSS questionnaires, there was no significant improvement at the 1-month assessment (ANOVA p> 0.05; **Figure 3**). Statistically and clinically significant improvements were noted at 3 and 6 months (ANOVA p< 0.05; OAB-Q and OABSS; **Figure 3**). Specifically, OAB-Q scores decreased from 69.5 ± 11.76 at baseline to 44.4 ± 12.06 at 6 months, and OABSS scores decreased from 9 ± 1.155 to 6.2 ± 2.394, indicating a substantial reduction in symptom burden and improvement in quality of life.

### 3.4 Patient satisfaction and tolerability

Assessment of satisfaction over time revealed different trends for therapeutic benefit and device comfort. Symptom improvement satisfaction was reported by 50% (5/10) of patients at 1 month, 60% (6/10) at 3 months, and 80% (8/10) at 6 months. Device comfort satisfaction was reported by 50% (5/10) of patients at baseline, 40% (4/10) at 3 months, and 60% (6/10) at 6 months.

### 3.5 Safety profile

The intervention arm showed an extremely safe profile during the 6-month intervention. There were no adverse events such as device-related complications, procedure sequelae, unanticipated device effects, serious adverse events, device malfunction, migration, or explantation events, which indicates the favorable safety profile of this novel neuromodulation device and the minimally invasive implantation procedure.

## 4 Discussion

This prospective, single-arm pilot study provides the first clinical validation of a domestically developed implantable tibial neuromodulation system in a Chinese cohort with refractory OAB and UUI. The key findings of this study are threefold: first, the device demonstrates significant efficacy in reducing UUI and urgency episodes, with a 90% responder rate at the 6-month endpoint; second, the therapeutic effect exhibits a delayed onset, with statistically significant improvements emerging at 3 months post-activation; and third, the device has an excellent safety profile with no device-related or procedure-related adverse events reported during the follow-up period. These findings not only confirm the potential of this domestically developed iTNS system as a viable third-line treatment for refractory OAB but also address the unmet clinical need for accessible, minimally invasive neuromodulation options in Chinese patients.

The delayed therapeutic onset observed in this study—with significant reductions in UUI episodes and improvements in quality of life emerging at 3 months rather than immediately post-activation—is consistent with the proposed neurophysiological mechanisms of tibial neuromodulation. Tibial nerve stimulation is thought to modulate bladder function through afferent pathways that project to the sacral and pontine micturition centers, inducing gradual reorganization of the central nervous system (CNS) and restoration of normal micturition control. Unlike pharmacotherapy, which may exert acute effects by directly targeting bladder smooth muscle or neurotransmitter receptors, neuromodulation requires time to induce plastic changes in the neural circuits regulating bladder function. This delayed response is consistent with findings from previous studies on PTNS and iTNS, where therapeutic effects typically become evident 2–3 months after the initiation of treatment (6–9). Clinically, this finding is important for patient counseling, as it emphasizes the need for long-term adherence to treatment to achieve optimal outcomes, and helps manage patient expectations regarding the timing of symptom improvement.

Clinically, the delayed therapeutic onset observed in this trial puts forward standardized patient perioperative management recommendations. Surgeons must fully inform patients of the expected 2–3 months lag period of efficacy before implantation, clarify that obvious symptom relief cannot be guaranteed within the first 3 months, and avoid premature judgment of treatment failure and early device removal. Regular follow-up visits within 3 months are required to adjust stimulation parameters to optimize early therapeutic response.

When compared to FDA-approved iTNS systems (eCoin™ and Revi™), the domestically developed device in this study offers several distinct advantages that enhance its clinical utility. The lead-less, ultra-compact cylindrical design (diameter 20.2 mm, thickness 4.8 mm) reduces the risk of device-related discomfort and improves cosmetic acceptability, which may enhance long-term patient adherence. In contrast, some existing iTNS devices have larger profiles or require lead placement, which can increase the risk of migration or local irritation. Additionally, the device’s high-efficiency power circuitry provides a projected battery lifespan of 3–5 years under default stimulation parameters, which is comparable to or longer than that of existing devices, reducing the need for frequent device replacement and associated surgical procedures. Critically, the automated, pre-programmed “set-and-forget” operation schedule eliminates the need for daily patient intervention, distinguishing it from both traditional PTNS (which requires weekly clinic visits) and some iTNS systems that require periodic reprogramming. This feature significantly reduces the treatment burden on patients and healthcare providers, potentially improving long-term adherence and clinical outcomes—evidenced by the 90% retention rate observed in this study.

The 90% responder rate at 6 months observed in this study compares favorably with established third-line therapies for refractory OAB. Sacral neuromodulation (SNM), which is considered a gold standard for third-line treatment, typically achieves responder rates of 60-80% for UUI, but carries substantial risks including lead migration (12%), surgical revision (30-40%), and infection (5-10%) (10–12). Intravesical botulinum toxin injection achieves similar responder rates but requires repeated injections every 3–6 months and carries a 5-30% risk of urinary retention, which may necessitate clean intermittent catheterization. In contrast, our study observed zero device-related adverse events, highlighting the safety advantage of the minimally invasive ankle approach, which avoids deep neurovascular structures and reduces the risk of complications associated with more invasive procedures like SNM. This favorable safety-efficacy profile makes the domestically developed iTNS system a particularly attractive option for patients who are unwilling or unable to undergo more invasive treatments, such as the elderly or those with comorbidities that increase surgical risk.

Another key advantage of this iTNS system is its ability to eliminate the daily treatment burden inherent in transcutaneous and percutaneous TNS approaches. Conventional PTNS requires 30-minute daily sessions with external devices, which can be time-consuming and inconvenient for patients, leading to poor long-term adherence (8, 9, 13). In contrast, the automated system in this study delivers continuous therapy without patient involvement after initial activation, directly contributing to the high protocol compliance rate observed. The integrated two-way visual programmer represents a generational advance over first-wave iTNS devices, enabling real-time monitoring of device parameters and battery status, as well as on-demand adjustments to optimize therapeutic efficacy. This feature enhances clinical flexibility, allowing healthcare providers to tailor treatment to individual patient needs and address any emerging issues promptly.

Notably, no significant reduction in nocturia was observed throughout the 6-month follow-up. This phenomenon can be explained by the therapeutic mechanism of iTNS and the multifactorial pathogenesis of nocturia. Implantable tibial neuromodulation primarily modulates peripheral bladder afferent nerve signals ascending to sacral pontine micturition centers to relieve bladder overactivity and daytime urgency incontinence. However, nocturia is jointly affected by nocturnal polyuria, sleep disorders, age-related renal concentration decline, mental anxiety and nighttime fluid intake, which cannot be fully intervened by single tibial nerve afferent regulation. Combined nocturia-targeted intervention (e.g., fluid restriction at night, sleep improvement) may be required for patients with prominent nocturia.

Voiding frequency was only significantly reduced at the 1-month follow-up without sustained efficacy at 3 and 6 months. In the early postoperative activation stage, continuous low-intensity tibial nerve stimulation rapidly suppresses acute hyper-excitability of bladder afferent nerves, thereby immediately lowering frequent voiding. With progressive central neural plastic remodeling over 3–6 months, the central micturition reflex gradually rebalances, and the early sharp reduction of voiding frequency partially rebounds, resulting in loss of statistical significance at later time points.

From a health economic and clinical popularization perspective, this domestically developed iTNS system has prominent advantages compared with imported neuromodulation devices. Independent domestic research and development greatly reduces equipment procurement and long-term maintenance costs, lowering the economic burden for Chinese patients. In addition, the fully automatic post-activation working mode eliminates repeated outpatient adjustment, which reduces the pressure of tertiary hospitals and is suitable for grassroots primary medical institutions to carry out third-line neuromodulation treatment for refractory OAB, showing broad grassroots clinical promotion potential.

The findings of this study also have important implications for the clinical management of refractory OAB. The delayed therapeutic onset suggests that iTNS should be considered as a long-term treatment option, rather than a short-term intervention, and patients should be counseled accordingly. The high responder rate and excellent safety profile indicate that this device may be suitable for a wide range of refractory OAB patients, including those who have failed other third-line treatments. Additionally, the improvements in patient-reported outcomes (OAB-Q and OABSS scores) and high satisfaction rates highlight the patient-centric benefits of this treatment, which extends beyond objective symptom reduction to improve overall quality of life.

The changing trend of device comfort satisfaction over follow-up reflects the process of patient postoperative tissue adaptation. The temporary decline in comfort satisfaction at 3 months is mainly caused by postoperative soft tissue edema, local foreign body sensation and intermittent nerve stimulation paresthesia. As subcutaneous tissue heals and patients gradually adapt to continuous nerve stimulation, foreign body discomfort is relieved at 6 months, leading to a rebound increase in device tolerance and satisfaction.

At present, the optimal standardized treatment protocol of percutaneous tibial nerve stimulation has not been established globally. Ergin et al. confirmed that the weekly application frequency of PTNS directly changes clinical efficacy; unstable outpatient attendance frequency will lead to fluctuating symptom relief effects (14, 15). Different from intermittent outpatient PTNS, implantable iTNS delivers continuous, stable neuromodulation according to preset parameters after one-time implantation, free from the influence of patient follow-up compliance and treatment frequency variation. This stable long-term stimulation mode is a core practical advantage of iTNS over traditional PTNS, which helps maintain sustained symptom improvement.

It is important to contextualize these findings within the broader landscape of OAB treatment. While iTNS offers significant advantages, it is not a panacea for all refractory OAB patients. Future studies should explore factors that predict treatment response, such as patient age, symptom severity, and comorbidities, to identify the subset of patients who are most likely to benefit from this therapy. Additionally, comparative studies with existing third-line treatments (e.g., SNM, botulinum toxin injection) are needed to further establish the relative efficacy and safety of this domestically developed iTNS system.

### 4.1 Limitations

This study has several limitations that should be considered when interpreting the results. First, the small sample size and exploratory pilot design limit the statistical power and generalizability of the findings. The single-arm, open-label nature of the trial introduces potential bias, as neither participants nor assessors were blinded to the intervention. Furthermore, the single-center setting may restrict the applicability of the results to broader clinical environments. Follow-up was limited to 6 months, which precludes conclusions regarding the long-term efficacy and safety of the device. Patient satisfaction was measured using a non-validated, study-specific questionnaire; although it captured meaningful patient-reported outcomes, its psychometric properties have not been formally established. Finally, the all-female cohort means the findings may not be generalizable to male patients. Future multi-center, randomized controlled trials with larger cohorts, longer follow-up, and validated patient-reported outcome measures are warranted to confirm and extend these preliminary results.

## Data Availability

The datasets presented in this study can be found in online repositories. The names of the repository/repositories and accession number(s) can be found in the article/**Supplementary Material**.

## Supplementary material

Supplementary Table 1 Mean daily UUI and urgency episodes across all follow-up timepoints (mean ± SD). P values for pairwise comparison marked within the table footnote in full manuscript.
