# Supplementary material for: Efficacy and safety of an implantable tibial neuromodulation system for overactive bladder with urgency urinary incontinence: an open-label, single arm trial
Source: Front Urol. 2026 Jul 15;6:1865760. doi: 10.3389/fruro.2026.1865760 (PMC13414830; doi:10.3389/fruro.2026.1865760)
Supplement: Supplementary Table 1 — Mean daily UUI and urgency episodes across all follow-up timepoints (mean ± SD). P values for pairwise comparison marked within the table footnote in full manuscript. [file Table1.docx]

**Supplementary Table S1 Mean daily UUI and urgency episodes across all follow-up timepoints (mean ± SD)**

| Outcome index | Baseline | 1 month | 3 months | 6 months |
| --- | --- | --- | --- | --- |
| Daily UUI episodes | 3.235 ± 1.14 | 2.612 ± 1.30 | 0.766 ± 0.69 | 0.632 ± 0.58 |
| Daily urgency episodes | 12.45 ± 4.21 | 10.12 ± 3.97 | 6.87 ± 3.05 | 5.31 ± 2.76 |

P values for pairwise comparison marked within the table footnote in full manuscript.
